# Crystal structures and photoluminescence characteristics of cesium lead bromide perovskite nanoplatelets depending on the antisolvent and ligand used in their syntheses

**DOI:** 10.1016/j.heliyon.2023.e23276

**Published:** 2023-12-07

**Authors:** Valdi Rizki Yandri, Adhita Asma Nurunnizar, Rima Debora, Priastuti Wulandari, Natalita Maulani Nursam, Rahmat Hidayat, Efi Dwi Indari, Yoshiyuki Yamashita

**Affiliations:** a Doctoral Program in Physics, Faculty of Mathematics and Natural Sciences, Institut Teknologi Bandung, Jl. Ganesha 10, Bandung 40132, West Java, Indonesia; b Physics of Magnetism and Photonics Research Division, Faculty of Mathematics and Natural Sciences, Institut Teknologi Bandung, Jl. Ganesha 10, Bandung 40132, West Java, Indonesia; c Research Center of Electronics, National Research and Innovation Agency, Jl. Sangkuriang, Bandung 40132, West Java, Indonesia; d Optical Ceramics Group, Research Center for Electronic and Optical Materials, 1-2-1 Sengen, Tsukuba, Ibaraki, 305-0047 Japan; e Nano Electronics Device Materials Group, Research Center for Electronic and Optical Materials, National Institute of Materials Science, 305-0044 1-1 Namiki Tsukuba Ibaraki, Japan; f Department of Applied Chemistry, Faculty of Engineering, Kyushu University, Japan; g Department of Electrical Engineering, Polytechnic State of Padang, Limau Manis Padang 25164, West Sumatra, Indonesia

**Keywords:** CsPbBr_3_, Halide perovskite, Photoluminescence, Nanocrystals, Ligand-assisted reprecipitation method, Exciton polaron

## Abstract

Cesium lead bromide (CsPbBr_3_) nanocrystals (NCs) with nanoplatelet shapes and different crystal structures were synthesized via the ligand-assisted reprecipitation (LARP) method using different pairs of ligands and antisolvents, namely oleic acid (OA) or linoleic acid (LA) as the ligand and toluene or chloroform as the antisolvent. The XRD data revealed that the obtained CsPbBr_3_ NCs have different crystal structures, namely orthorhombic, tetragonal, and cubic, depending on the ligand and antisolvent pair, which exhibited significantly different photoluminescence (PL) characteristics. From the XPS data, these CsPbBr_3_ nanoplatelets showed two doublet peaks of the Br-3d orbital at different binding energies, representing two different chemical environments of the Br bonds. The doublet peak apparent at a higher binding energy was associated with the Br chemical states at the crystal surface, which appeared because of the distorted crystal structure resulting from the interaction of the solvent and ligand with Br ions. The PL emission consists of three luminescence centers: a PL band peaked at 520 nm (A band), a PL band peaked at 540 nm (B band), and a PL band tail, which can be discussed in terms of exciton models. Stable and intense luminescence was observed in CsPbBr_3_ nanoplatelets synthesized using a pair of toluene antisolvent and LA ligand, namely CsPbBr_3_#(Tl/LA). The orthorhombic crystal structure and distorted crystal surface in this sample may lead to confinement of the photogenerated small exciton-polaron and weak phonon interactions, which effectively hinder exciton dissociation, particularly at the crystal surface, resulting in intense PL. The results of this study may provide additional important insights into the role of the antisolvent and ligand in the formation of CsPbBr_3_ NCs and the exciton behavior in their PL characteristics, which may also be found in other types of halide perovskites.

## 1 Introduction

Hybrid organic-inorganic halide perovskite materials (ABX_3_, where A is an organic cation, B is Pb^2+^ or Sn^2+^, and X is a halide anion) have attracted considerable attention for various applications. Methyl ammonium lead iodide (MAPbI_3_) is the most studied halide perovskite, which was initially aimed for solar cell applications. For further improvements, many efforts have been made to modify this halide perovskite by replacing A and X ions with various possible cations and anions. Halide perovskites offer several advantages as active materials in solar cells because of their low production cost [1], facile solution-based synthesis [2], and excellent optical absorption for efficient solar energy conversion [3]. To date, these perovskite materials have shown a wide range of potential applications, not only for solar cells [4] but also for light-emitting devices [5,6], lasers [7], photodetectors [8], etc. [9]. This fascinating progress has stimulated other efforts to investigate all-inorganic perovskite materials such as CsPbBr_3_, CsPbCl_3_, and CsPbI_3_ [[10], [11], [12]]. Although these all-inorganic perovskites cannot produce solar cells with conversion efficiencies exceeding those of their MAPbI_3_ counterparts, their photoluminescence (PL) characteristics have attracted considerable attention for light-emitting device applications [13].

All-inorganic perovskites can be synthesized using various methods, such as hot injection [14], ligand-assisted reprecipitation (LARP) [15], ultrasonication [16], and indirect (two-step) synthesis [17]. In the LARP method, CsBr and PbBr_2_ are dissolved in a solvent to form a CsPbBr_3_ precursor solution, in which ligands are added to control the formation of perovskite NCs [[18], [19], [20]]. An antisolvent was dripped into the precursor solution to induce fast (or intermediate) precipitation of perovskite NCs [21]. Various antisolvents have been reported for NCs synthesis, including diethyl ether [22], chlorobenzene [23], chloroform [24], toluene [25], and ethanol [26]. However, the stability and degradation of CsPbX_3_ NCs remain important issues that need to be resolved. CsPbX_3_ NCs are formed via ionic bonding, which can easily dissociate upon exposure to moisture, heat, and light [27]. Several studies have been conducted to find a method for improving the crystal stability and photoluminescence characteristics by modifying the ligands [[28], [29], [30]]. Other efforts have also been made to suppress NCs degradation by impregnation into polymers or porous media, and coating with SiO_2_ or TiO_2_ [31,32].

In addition to their stability, the PL characteristics of CsPbX_3_ NCs also remain a matter of concern. Various CsPbX3 NCs structures with different degrees of dimensionality from three-dimensional (3D) to zero-dimensional (quantum dot) structures have been reported [[33], [34], [35]]. 3D bulk CsPbBr_3_ NCs are the most stable, but exhibit weak PL [36]. In general, bulk CsPbX_3_ may have different crystal structures, that is, orthorhombic, tetragonal, or cubic, depending on the temperature [37,38] and ligands used in their synthesis [35,39]. The orthorhombic structure is stable at temperatures lower than 88 °C, while the tetragonal structure is stable at temperatures between 88 °C and 130 °C, whereas the cubic structure is stable at temperatures higher than 130 °C. Strong PL of CsPbBr_3_ quantum dots has been reported in the range of 420–520 nm, with the quantum dot size ranging between 3.8 and 11.8 nm [37]. The quantum size confinement effect plays an important role in this structure, resulting in intense PL emission [40]. In general, to synthesize CsPbBr_3_ quantum dots, oleic acid and oleylamine are used as ligands or capping agents to encapsulate and stabilize the formed quantum dots [[41], [42], [43]]. Several studies have reported that varying the oleylamine-to-oleic acid ratio may considerably affect the morphology and size of CsPbX_3_ NCs [18,28]. The use of ligands such as didodecyl dimethylammonium bromide and sodium dodecylbenzene sulfonate can improve the stability of CsPbBr_3_ NCs [44], indicating the important role of ligands during crystallization and thereafter. However, the effects of ligands and antisolvents on the resulting PL characteristics have mostly been linked to NC morphology and size evaluation, whereas the correlation between these ligand-antisolvent effects and crystal structures, crystal shape dimensionality, and photoluminescence has rarely been explored.

Herein, we report the synthesis of CsPbBr_3_ NCs at room temperature via the ligand-assisted reprecipitation (LARP) method in the presence of a single ligand (either LA or OA) as a capping agent and correlate their PL characteristics with the formed crystal structures. In the absence of oleylamine (OlAm), the ligands and antisolvents used in this study promoted the formation of 2D or nanoplatelet-like CsPbBr_3_ NCs. The present study demonstrates that the synergetic effects of the ligand and antisolvent play an essential role in determining the crystal structure of these nanoplatelets, which considerably affects their PL characteristics.

## 2 Experimental

### 2.1 CsPbBr_3_ nanocrystal synthesis and thin layer preparation

The precursors of CsPbBr_3_ perovskite materials, namely CsBr (>99.0 %) and PbBr_2_ (>98.0 %), were purchased from Tokyo Chemical Industry Co. Ltd. and used as it is. LA and OA ligands were purchased from Sigma-Aldrich and were used as it is. Dimethylsulfoxide (DMSO) and dimethylformamide (DMF) were used as precursor solvents in the synthesis of CsPbBr_3_ NCs, whereas toluene and chloroform were used as antisolvents. All solvents were of analytical grade (p.a.) and purchased from Merck.

**Fig. 1:**
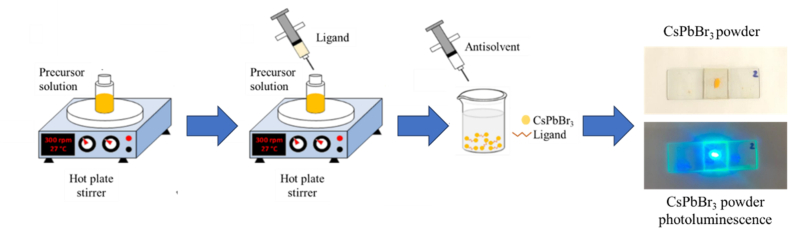
Schematic illustration of the experimental steps for CsPbBr_3_ NCs synthesis via the LARP method. The resulting CsPbBr_3_ NCs powder exhibited bright photoluminescence upon green-pointer laser illumination. (For interpretation of the references to color in this figure legend, the reader is referred to the Web version of this article.)

The synthesis process was carried out in sequential multistep, as shown in Fig. 1. The 0.5 M precursor solution of CsPbBr_3_ was prepared by dissolving CsBr and PbBr_2_ in a DMF:DMSO (1:1 v/v) solvent mixture. The LA or OA ligand was then added to the precursor solution at a ligand-to-precursor solvent concentration ratio of 1:2. The solution was subsequently stirred for 60 min. An antisolvent, either chloroform or toluene, was then added to the solution and stirred for 1 h. All the synthesis steps were conducted at room temperature. After the CsPbBr_3_ NCs precipitated, the precipitate was washed and dried by heating to 190 °C under vacuum until the remaining solvent was completely evaporated. To measure the absorption spectra, a colloidal solution of CsPbBr_3_ was spin-coated onto a TiO_2_ mesoporous layer and heated to 190 °C under vacuum to evaporate residual solvents.

The synthesized samples could be separated into two groups depending on the antisolvent and ligand used during sample preparation, as shown in Table 1.

**Table 1:** CsPbBr_3_ NCs synthesized via the LARP method.

No.	Sample name	LARP method			
		Antisolvent		Ligand	
		Toluene (Tl)	Chloroform (Cl)	Oleic Acid (OA)	Linoleic Acid (LA)
1.	CsPbBr_3_#(Tl/LA)	×			×
2.	CsPbBr_3_#(Tl/OA)	×		×	
3.	CsPbBr_3_#(Cl/LA)		×		×
4.	CsPbBr_3_#(Cl/OA)		×	×	

### 2.2 Characterizations

X-ray diffraction (XRD) patterns were measured by using an X-ray diffractometer (Bruker D8 Advance) at a scan rate of 10° min^−1^. The PL spectra of CsPbBr_3_ NCs samples were measured using a fluorescence spectrophotometer (Hitachi F-2700 FL 2957-004). The nanoscale morphology and shape of the NCs were characterized using high-resolution transmission electron microscopy (HRTEM) (Hitachi H-9500). In addition, High-resolution X-Ray Photoelectron Spectroscopy (XPS) measurements were performed using PHI Quantes (ULVAC-PHI).

## 3 Results and discussion

### 3.1 Crystal structures of CsPbBr_3_ NCs

**Fig. 2:**
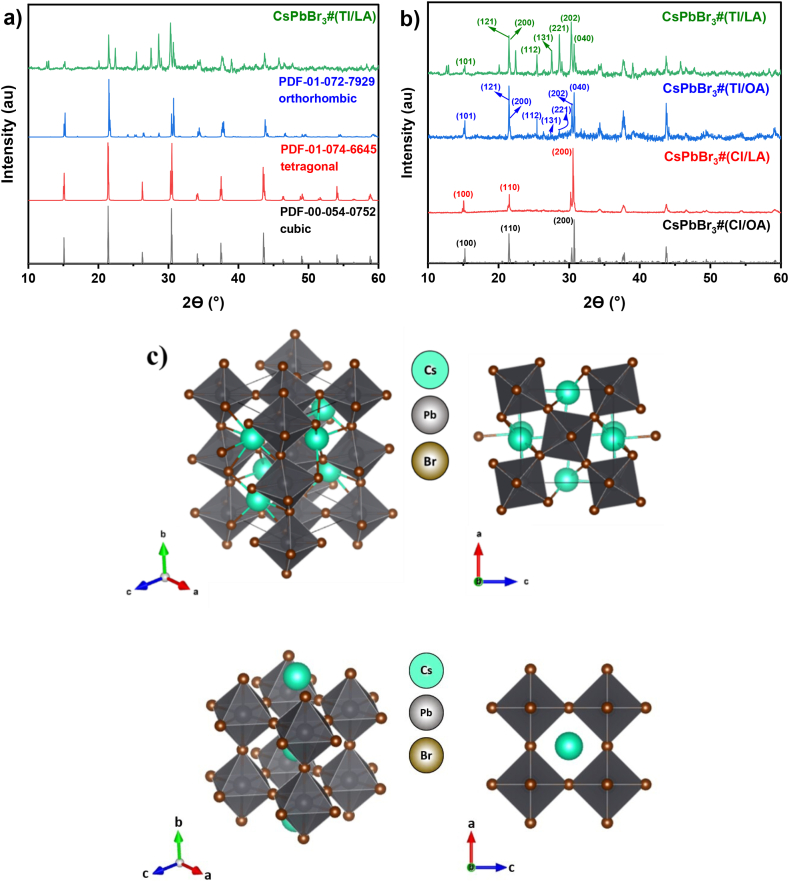
(a) XRD patterns of CsPbBr_3_#(Tl/LA) NCs and the reference XRD data for orthorhombic CsPbBr_3_ (PDF-01-072-7929), cubic CsPbBr_3_ (PDF-00-054-0752), tetragonal CsPbBr_3_ (PDF-01-074-6645). (b) XRD patterns of CsPbBr_3_#(Tl/LA), CsPbBr_3_#(Tl/OA), CsPbBr_3_#(Cl/LA), and CsPbBr_3_#(Cl/OA) NCs. (c) The orthorhombic crystal structure of CsPbBr_3_ with *Pnma* space group symmetry drawn from the XRD data in Figure (a). (d) The cubic crystal structure, as a comparison to show the difference in octahedral tilting and orientation with those in other crystal structures.

Fig. 2(a) shows the XRD patterns of the CsPbBr_3_ NCs synthesized using toluene as the antisolvent and LA as its ligand (CsPbBr_3_#Tl/LA). Several prominent peaks were observed at 15.2°, 21.5°, 21.6°, 30.3°, and 30.7°, corresponding to the (101), (121), (200), (040), and (202) planes, respectively. The XRD patterns of all CsPbBr_3_ samples were analyzed by comparing them with the powder diffraction files (PDF) no. 01-072-7929, no. 01-074-6645, and no. 00-054-0752, which represent the reference diffraction data for orthorhombic CsPbBr_3_, tetragonal CsPbBr_3_, and cubic CsPbBr_3_, respectively. The CsPbBr_3_#(Tl/LA) NCs were found to have an orthorhombic crystal structure with *Pnma* space group symmetry and lattice constants of *a* = 8.21 Å, *b* = 11.74 Å, and *c* = 8.26 Å (Fig. 2(c)). This result is nearly identical to the experimental results of CsPbBr_3_, reported by Hong et al. (*a* = 8.26 Å, *b* = 11.76 Å, and *c* = 8.21 Å) [45] and Atourki et al. (*a* = 8.25 Å, *b* = 11.70 Å, and *c* = 8.21 Å) [46]. Moreover, the result obtained herein is similar to the computational results reported by Tomanová et al. (*a* = 8.38 Å, *b* = 11.49 Å, *c* = 7.62 Å) [47] and Lianburg et al. (*a* = 8.25 Å, *b* = 11.75 Å, *c* = 8.20 Å) [48]. On the other hand, the XRD peaks of CsPbBr_3_#(Cl/LA) and CsPbBr_3_#(Cl/OA) NCs were found to have a cubic crystal structure with Pm-3m space group symmetry (Fig. 2(d)) and lattice constant of *a* = *b* = *c* = 5.89 Å and 5.87 Å, respectively. These XRD peaks are similar to other experimental results for cubic CsPbBr_3_ reported by Sun et al. [49].

To analyze the crystal structures of these four different samples, their XRD patterns were compared with the PDF reference for the orthorhombic, cubic, and tetragonal phases of CsPbBr_3_ (Fig. 2(a)). The XRD patterns of the other samples were remarkably different, as clearly seen in Fig. 2(b). The XRD pattern of CsPbBr_3_#(Tl/LA)) shows the most distinct characteristics, indicated by a strong peak at 25.4°, accompanied by several peaks between 22° and 30°, which is a typical XRD pattern of orthorhombic CsPbBr_3_ crystals. The CsPbBr_3_#(Tl/OA) sample showed a strong peak at 25.4° without peaks between 22° and 30°, which is a typical XRD pattern of tetragonal CsPbBr_3_ crystals. In contrast, the samples prepared using the chloroform antisolvent do not show peaks between 22° and 30°, indicating that both NCs have a cubic structure. The results of the XRD analysis using Match software are listed in Table 2. The formed CsPbBr_3_ crystal structure is critically determined based on the antisolvent and ligand used in the synthesis.

**Table 2:** Crystallographic data for CsPbBr_3_ NCs synthesized using different ligands and antisolvents.

CsPbBr_3_ NCs	*a* (Å)	*b* (Å)	*c* (Å)	*V* (Å^3^)	Space group	Crystal structure
CsPbBr_3_#(Tl/LA)	8.21	11.74	8.26	795.98	*Pnma*	Orthorhombic
CsPbBr_3_#(Tl/OA)	5.81 (*a* = *b*)		5.86	197.96	*P*4*mm*	Tetragonal
CsPbBr_3_#(Cl/LA)	5.89 (*a* = *b* = *c*)			203.29	Pm-3m	Cubic
CsPbBr_3_#(Cl/OA)	5.87 (*a* = *b* = *c*)			202.71	Pm-3m	Cubic

**Fig. 3:**
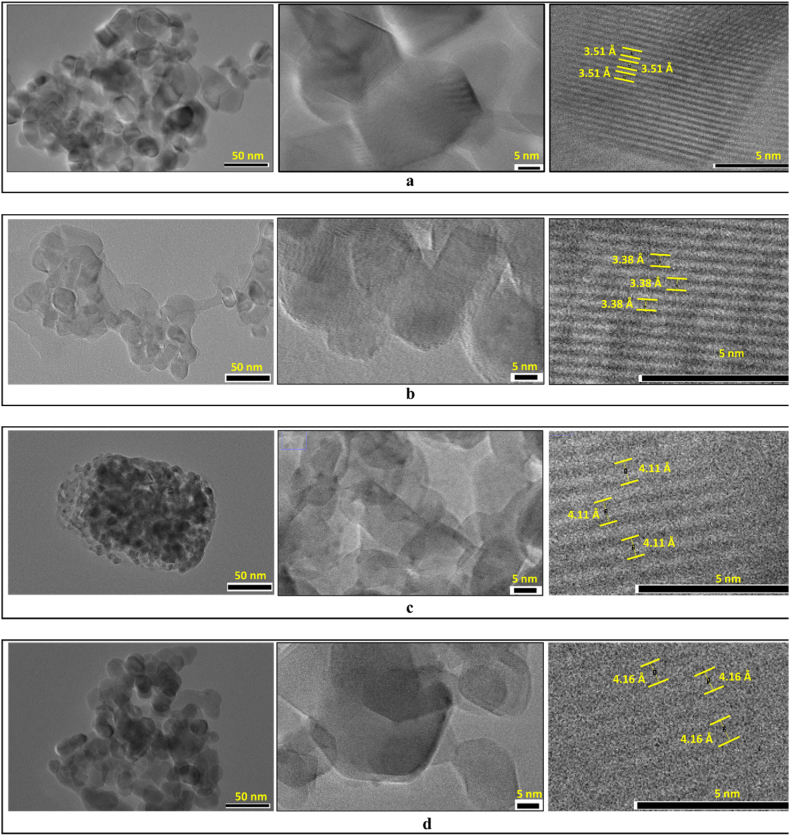
HR-TEM image (left) and its Miller indices (*d*_*hkl*,_ right) for (a) CsPbBr_3_#(Tl/LA) with *d*_211_ = 3.51 Å, (b) CsPbBr_3_#(Tl/OA) with *d*_111_ = 3.38 Å, (c) CsPbBr_3_#(Cl/LA) with *d*_110_ = 4.11 Å and (d) CsPbBr_3_#(Cl/OA) with *d*_110_ = 4.16 Å.

HR-TEM measurements were conducted to further confirm the crystal shape and structure of all CsPbBr_3_ samples, where the *d*-spacing for a particular diffraction plane could also be observed and determined directly from these HR-TEM images. The HRTEM image is shown in Fig. 3, along with the measured lattice constants of the CsPbBr_3_ NCs. Fig. 3(a) shows that CsPbBr_3_ synthesized using LA forms a randomly stacked nanoplatelet-like shape consisting of only a few layers, where the lattice pattern from the lower sheet can still be seen to overlap with the lattice pattern from the upper sheet. The observed lattice pattern agrees with the diffraction pattern of the (211) plane with *d*_211_ = 3.51 Å for the orthorhombic crystal structure shown in Table 2. As shown in Fig. 3(b), the NCs prepared using OA also exhibited nanoplatelet-like shapes. The observed lattice pattern is from the (111) plane of the tetragonal structure, where *d*_111_ = 3.38 Å. In contrast, in Fig. 3(c) and (d), the observed lattice pattern periodicity is ∼4.11 Å and ∼4.16 Å, which can be assigned to diffraction from the (110) plane of the cubic structure. The orthorhombic crystal structure is shown in Fig. 2(c), where the octahedral PbBr_6_ structures are slightly tilted and rotated, displacing the Br anions from their ideal positions in the cubic structure (Fig. 2(d)).

Toluene is an aromatic hydrocarbon with low polarity (0.099), whereas chloroform is a non-aromatic solvent with high polarity (0.259) [50,51]. Polarity is related to the solvation capability (or solvation power) of the perovskite precursor ions and the ligand, and the interactions of ions and ligands. Here, the polarity of the antisolvent may affect the NCs formation process. Moreover, toluene and chloroform have a large difference in their boiling points. Toluene has a higher boiling point (110.6 °C) than chloroform (61.2 °C). Therefore, toluene can take a longer time to influence the crystallization process until all the solvents evaporate completely. It has been reported elsewhere that CsPbBr_3_ NCs may have three different structural phases: cubic (Pm-3m), tetragonal (*P*4*mm*), and orthorhombic (*Pnma*) [[52], [53], [54]]. The crystal structure of CsPbBr_3_ at room temperature is orthorhombic, which can transform into tetragonal and cubic structures after heating to 88 °C and 130 °C, respectively. However, the different ligands and antisolvents used during the synthesis yielded different final NCs structures, despite the synthesis being carried out at the same process temperature. The synergetic effect between the toluene antisolvent and ligand resulted in nanoplatelets with different crystal structures, namely an orthorhombic crystal structure for the LA ligand and a tetragonal crystal structure for the OA ligand. On the other hand, chloroform tended to produce nanoplatelets with a cubic structure regardless of the ligand used. The LA ligand effectively interacts with the cation in the toluene antisolvent, resulting in an extensive chain conformation, in which the C=C bond in the LA ligand causes a chain bending of ∼60°, in contrast to the chain bending of just ∼30° in the OA ligand. In this case, during NCs formation, owing to their bulky chains, LA ligands are attached to the crystal surface with a larger and more random anchoring angle, leading to the favorable formation of an orthorhombic structure rather than cubic or tetragonal structures.

### 3.2 Photoluminescence characteristics of CsPbBr_3_ NCs

**Fig. 4:**
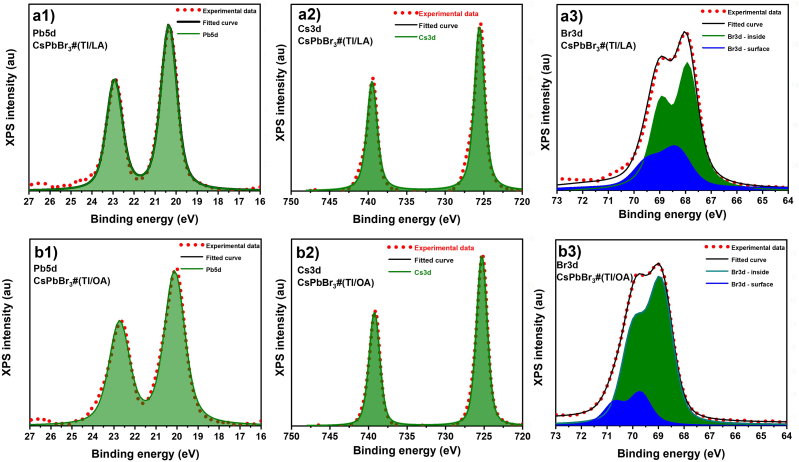
XPS spectra of the Pb-5d, Cs-3d and Br-3d orbitals for (a1-a3) CsPbBr_3_#(Tl/LA) and (b1-b3) for CsPbBr_3_#(Tl/OA).

Before discussing their PL characteristics, we should analyze beforehand the XPS data of these nanoplatelet samples. Fig. 4 shows the XPS spectra of Pb-5d, Cs-3d and Br-3d orbitals for CsPbBr_3_#(Tl/LA) and for CsPbBr_3_#(Tl/OA) samples. The XPS peaks of Pb-5d for those samples are almost similar, which can be fitted with a single Voigt function. These peaks can be assigned to d^5/2^ and d^3/2^ spin–orbit coupling with a peak area ratio of 1.5. The XPS peaks of Cs-3d orbital for those samples are also similar. However, as evident in Fig. 4 (a3) and (b3), the XPS peaks of the Br-3d orbital of the two samples are different and must be fitted with two doublet Voigt functions at different apparent binding energies. The CsPbBr_3_#(Tl/LA) sample has a larger portion of the higher-energy doublet compared to the CsPbBr_3_#(Tl/OA) sample. This may indicate a higher non-uniformity of the chemical states of Br in the CsPbBr_3_#(Tl/LA) sample, which may be related to the greater crystal structure distortion at the crystal surface caused by the ligand and antisolvent during nanocrystal formation. Owing to the larger bending angle of the LA ligand and its interaction with Br anions through its acid group, the distortion of the Br–Pb and Br–Cs bonds on the nanocrystal surface increases, causing the appearance of this Br-3d doublet peak with a higher binding energy.

**Fig. 5:**
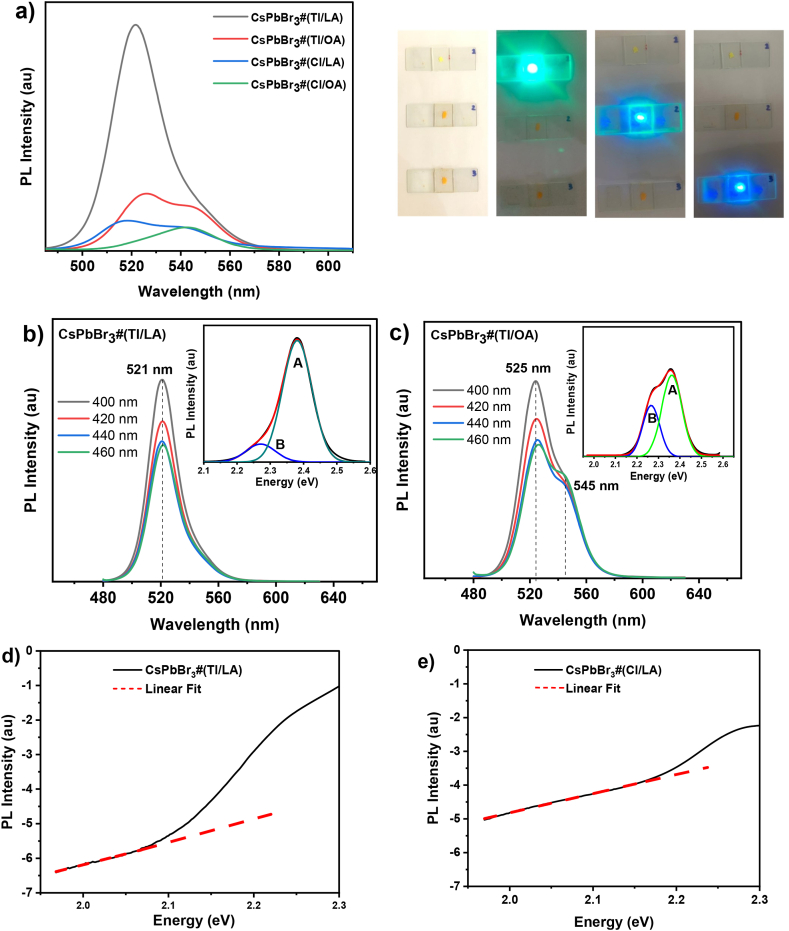
(a) PL spectra of CsPbBr_3_ NCs synthesized using different ligands and antisolvents. The photograph shows the CsPbBr_3_#(Tl/LA), CsPbBr_3_#(Tl/OA) and CsPbBr_3_#(Cl/LA) samples (from top to bottom) when were illuminated by a blue pointer laser. (b) PL spectra of CsPbBr_3_#(Tl/LA) NCs powder at various excitation wavelengths in the range of 400–460 nm. (c) PL spectra of CsPbBr_3_#(Tl/OA) NCs powder. The inset figures show the fitting results of the spectra with two Gaussian functions, labeled as the A and B peaks. The PL band tail characteristics in CsPbBr_3_ NCs prepared with LA ligand and toluene (d) or chloroform (e) antisolvent. The curve fitting lines of these Urbach band tails are indicated by the red dashed line. (For interpretation of the references to color in this figure legend, the reader is referred to the Web version of this article.)

The PL spectra of the CsPbBr_3_ NC powders are shown in Fig. 5(a), where the PL spectral shape and intensity vary depending on the ligand and antisolvent. photograph shows the appearance of PL emission when the CsPbBr_3_#(Tl/LA), CsPbBr_3_#(Tl/OA), and CsPbBr_3_#(Cl/LA) samples (from top to bottom) were illuminated by a blue pointer laser, showing a remarkable difference in their PL brightness by the naked eye. Fig. 5(b) shows the PL spectrum of CsPbBr_3_#(Tl/LA); however, it exhibited a single narrow peak at ∼521 nm with a width of 25 nm. The PL spectrum did not change with PL excitation wavelength in the measurement range of 400–460 nm. It should be noted that even without oleylamine, this PL characteristic is similar to that of CsPbBr_3_ synthesized with oleylamine, as reported elsewhere [28]. In contrast, the PL spectra of the other samples are composed of two PL peaks centered at 520–525 nm and 545 nm, whose intensities are much lower than those observed in the CsPbBr_3_#(Tl/LA) sample. The curve-fitting results of those PL spectra with two Gaussian functions show the presence of two PL bands peaked at 525 nm (labeled as the A band) and 545 nm (labeled as the B band). Fig. 5(c) shows the PL spectrum of the CsPbBr_3_ sample prepared using the OA ligand and a chloroform antisolvent. The fitting results in the inset figure show that the B-band intensity is proportional to the A-band intensity, where the A-band in this sample is much lower than that in the CsPbBr_3_#(Tl/LA) sample. The PL peak at 520 nm (A band) has been much reported in CsPbBr_3_ quantum dots [18,28,32] but not the peak at 542 nm (B band). This B-band does not originate from ligands or other impurities because LA and OA do not produce luminescence in this wavelength region [29]. Samples prepared using toluene consistently produced a higher PL intensity and a larger proportion of the A band than those prepared using chloroform.

Generally, PL characteristics are a manifestation of electronic processes and charge–carrier dynamics. At the present stage, we simply consider that the appearance of the two PL peaks originates from two different independent PL sites. It does not originate from either multistep excited-state relaxation or an energy-transfer process. The first PL peak (peak A at 520 nm) was predominantly present in orthorhombic CsPbBr_3_. On the other hand, the second PL peak (peak B at 545 nm) was predominantly present in the tetrahedral and cubic structures but weakly present in the octahedral structures, as shown by the curve-fitting lines in Fig. 5 (b) and (c). The PL spectra then indicate the presence of two or three different crystal structures in each sample, but CsPbBr_3_#(Tl/LA) NCs have the largest content of orthorhombic crystal structure, while CsPbBr_3_#(Cl/OA) NCs have the largest content of cubic crystal structure. The PL in CsPbBr_3_ quantum dots has been widely associated with excitons, which is indicated by the appearance of a narrow sharp peak in the absorption spectrum [55,56]. In general, as observed in silicon-based semiconductors, exciton states are formed at energy levels below the conduction band. Excitons can be formed and stabilized when the photogenerated electron–hole pair can support its interaction via Coulomb forces. These conditions can be achieved at low temperatures, leading to diminished thermal energy, or by quantum size confinement effects, such as in quantum dots. There have been many reports on the intense PL of CsPbBr_3_ in quantum dot shapes with cubic crystal structures. However, increasing the crystal size beyond the quantum dot size causes excitons to dissociate spontaneously, resulting in a separate electron-hole pair and a weakened PL emission. In the case of CsPbBr_3_#(Tl/LA) NCs, despite the NCs size observed in the HR-TEM image being much larger than the quantum dot size, intense PL was still observed.

Recently, many studies have highlighted that excitons in halide perovskites have different characteristics from those in conventional III–V semiconductors, which are formed via covalent bonding. Halide perovskites are predominantly formed via ionic bonding, although they also possess minor covalent bonding characteristics. Halide perovskites have a soft and polar lattice that can be easily coupled with phonon modes. Photoexcitation creates excitons that are coupled with lattice vibrations or phonons to form polaronic states, particularly in 2D perovskites [57,58]. Polarons are quasiparticles that are formed when a charged particle (e.g., an electron) interacts with its surroundings, causing lattice distortions. An exciton can dissociate into positive and negative polarons, which are separated hole and electron individually dressed by the lattice distortion surrounding them. In some cases, bipolarons may also be formed in which two opposite polarons are bound to each other by Coulomb force interactions.

However, the most probable excitons and their polaronic states in halide perovskites remain under extensive investigation. Free Wannier-like excitons are not favorable because of the soft and polar lattice characteristics of halide perovskites. The formation of large polarons in halide perovskites can explain the long lifetime of charge carriers but low carrier mobility [59]. Large polarons are often spread over several unit cells owing to weak long-range Coulomb interactions and small lattice distortions. However, small polarons with highly localized wave functions have also been reported for halide perovskites. Computational studies on halide perovskite electronic structures have demonstrated that the photoexcitation process mainly involves the 4*p* orbitals of Br^−^ in the valence band and the 6*p* orbitals of Pb^2+^ in the conduction band [60,61]. Notably, the orthorhombic crystal structure exhibits a low degree of symmetry owing to the tilting and rotation of the octahedral PbBr_6_ structures, as shown in Fig. 2(c). In contrast, the cubic crystal structure exhibits a high degree of symmetry without octahedral PbBr_6_ distortion, as shown in Fig. 2(d) [62]. Therefore, in the orthorhombic structure, the exciton is likely coupled with octahedral PbBr_6_ distortions, thereby transforming into self-trapped excitons (STEs) localized inside the sublattice CsPbBr_3_, which may also be identified as a small polaron. In addition, for the sample with the orthorhombic crystal structure prepared here, namely the CsPbBr_3_#(Tl/LA) sample, it has more crystal distortion on the surface, which hinders exciton diffusion and exciton dissociation at the crystal surface. This may then explain the appearance of a stronger PL emission in this sample than in the other samples.

The PL spectrum of the CsPbBr_3_#Tl/LA sample appears to have an asymmetric shape with a band tail at long wavelengths (i.e., low energy). However, as shown in the inset Fig. 5(b), this PL spectrum is composed of two different PL bands peaked at 2.27 and 2.38 eV (equivalent to peak B at 546 nm and peak A at 521 nm, respectively). In this sample, the proportion of the A band peaked at 2.38 eV was more dominant than that of the B band. On the other hand, PL samples comprised of two PL bands exist with the peak B at 2.27 eV being more dominant than the peak A. However, the second peak was much more prominent in the CsPbBr_3_ NC with a cubic crystal structure rather than in the CsPbBr_3_ NC with an orthorhombic crystal structure. Because the cubic structure has high symmetry, the crystals may be strongly coupled with the phonon mode, which spatially extends to several unit cells, leading to Fröhlich-like large polarons with dominant long-range interactions. In 3D crystals, the phonon mode density may not be substantially different for cubic or orthorhombic structures. However, this might not be entirely valid for 2D structures such as the CsPbBr_3_ NCs synthesized herein, where the exciton-phonon interaction in tetragonal perovskites is weaker because the lattice is softer than that in bulk perovskites. In CsPbBr_3_#Tl/LA, which forms orthorhombic with low crystal symmetry, the phonon mode density may be smaller than NCs with a cubic crystal structure. Therefore, as revealed in the present data, a local distortion or small exciton polaron is highly preferable in nanoplatelets NCs with an orthorhombic crystal structure. However, the other CsPbBr_3_ samples formed tetragonal and cubic crystal structures, which may possess a high phonon density. The formation of large exciton polaron, which can then dissociate into two separate polarons, leads to a substantially low probability of radiative recombination resulting in a substantially low PL intensity.

In addition to these two types of excitons, some previous studies have also reported unusual behavior of polaronic states caused by local structural dynamic disorders, such as momentarily trapped exciton polarons [63]. Computational and experimental analyses in these studies reported that the A cation may undergo fast rotation, while the sublattice PbBr_6_ octahedral structure may undergo deformation and rotation after photoexcitation, leading to a local dynamic structural disorder [[64], [65], [66], [67]]. In addition, several computational studies have also shown the presence of soft phonons, which mainly occur at the perovskite crystal surface, leading to local structural instability. This instability may trap primary photoexcited states in low-energy STEs before relaxing to the ground state by emitting photons during the PL process. However, it may also liberate cations (A^+^, B^+^) or anions (X^−^), especially in the presence of an internal electric field, thereby degrading the perovskite layer in solar cells. This type of disordered exciton may be apparent as a band tail in the absorption and PL spectra, which is known as the Urbach tail. The PL intensity decays exponentially as follows: $L\;\left(E,T\right)\propto \exp\left[\frac{\left(\sigma -1\right).\;E}{k_{B}\;.\;T}\right]$ (where *L* is the PL intensity, *E* is the photon energy, *T* is the temperature, *σ* is the steepness coefﬁcient, and *k*_*B*_ is the Boltzmann constant) [68]. This can be seen as a linear line in the PL spectrum for the *y*-axis on a logarithmic scale. Fig. 5(d) and (e) show that the slopes of the fitting lines are nearly the same, leading to similar *σ* values of 1.14 for CsPbBr_3_#Cl/LA and 1.16 for CsPbBr_3_#Tl/LA. Similar values were also found for the other samples, indicating the formation of STE owing to the state disorder at the band edge. However, the proportion of this PL is extremely small compared with that of the small and large exciton polarons in CsPbBr_3_#Tl/LA.

**Fig. 6:**
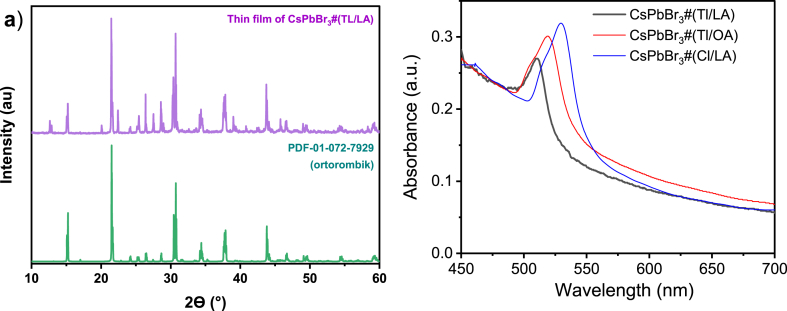
(a) XRD patterns of CsPbBr_3_#(Tl/LA) thin layer on TiO_2_ mesoporous substrate, showing similar orthorhombic structure as in its powder form. (b) Absorption spectra of CsPbBr_3_ NCs thin layer on TiO_2_ mesoporous substrate.

In order to confirm the formation of excitons in these samples, thin-layer samples of CsPbBr_3_ were then deposited by spin-coating the dispersion of CsPbBr_3_ NCs onto a mesoporous (mp-) TiO_2_ layer in an N_2_ atmosphere. The XRD pattern of the CsPbBr_3_#(Tl/LA) sample is presented in Fig. 6(a), which also shows an orthorhombic structure similar to the powder form. Because this sample forms a thin layer, although there is a strong light-scattering effect from the TiO_2_ layer, its absorption spectrum can still be measured, as shown in Fig. 6(b). A narrow sharp absorption peak appeared at approximately 512 nm (≈2.42 eV), 519 nm (≈2.39 eV), and 529 nm (≈2.34 eV) for CsPbBr_3_#(Tl/LA), CsPbBr_3_#(Tl/OA), and CsPbBr_3_#(Cl/LA), which has orthorhombic, tetragonal, and cubic crystal structures, respectively. These peaks can be assigned to the exciton peaks as mentioned above, which have different energies depending on the crystal structure. The absorption band edge also shifted to longer wavelengths (lower photon energy) in the order of orthorhombic, tetragonal, and cubic crystal structures, that is, with decreasing PbBr_3_ octahedral tilting. Several studies have reported that increasing the octahedral tilting with respect to its cubic structure increases the energy gap [69,70]. Similar to their PL peaks, the absorption exciton peaks appear to be composed of two peaks for CsPbBr_3_#(Tl/OA) and CsPbBr_3_#(Cl/LA), although the longer-wavelength peak is much more dominant. As mentioned above, the PL peaks in Fig. 5 are at 521 nm (≈2.38 eV), 525 nm (≈2.35 eV), and 545 nm (≈2.28 eV) for CsPbBr_3_#(Tl/LA), CsPbBr_3_#(Tl/OA), and CsPbBr_3_#(Cl/LA), respectively. By comparing these absorption and PL peaks, the energy difference of the absorption peaks and PL peaks are about ∼0.04–0.06 eV, where the cubic structure has the largest difference of 0.06 eV. These differences are related to the exciton binding energy. Therefore, in CsPbBr_3_#(Tl/LA), which shows the highest energy levels of the absorption and PL peaks, the photoexcited states are favorably formed as small polaronic excitons. In contrast, in the CsPbBr_3_#(Cl/LA) sample, the lower energy of the absorption and PL peaks indicates that large polaronic excitons are formed.

**Fig. 7:**
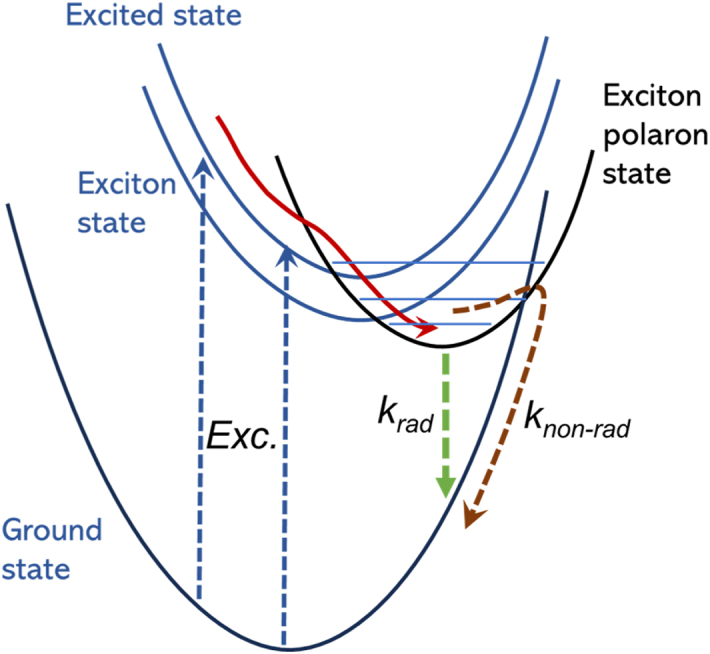
A configuration coordinate diagram schematically represents the role of the exciton polaron state in the relaxation of the excited state (represented by the red line path), resulting in a radiative process or PL (represented by the green line path) in these CsPbBr_3_ NCs. Non-radiative relaxation (represented by the brown dashed line) may also occur due to coupling with crystal vibrations or phonons, which becomes a competitive pathway for the radiative decay pathway at different ratios depending on the crystal structure. . (For interpretation of the references to color in this figure legend, the reader is referred to the Web version of this article.)

The detailed PL mechanism for explaining the origin of the PL characteristic difference cannot be explained comprehensively based only on the present experimental data without the ultrafast spectroscopic data of photoexcitation kinetics. However, the PL mechanisms in these samples may be explained using a modified configuration coordinate diagram for the self-trapped excitons [71]. As illustrated in Fig. 7, the photoexcited electron relaxes to the exciton polaron state (represented by the red line path) before finally returning to the ground state via a radiative process by emitting photons (PL). However, the photoexcited electron can also relax back to the ground state via a non-radiative pathway (represented by the brown line path), which may occur due to exciton coupling with lattice vibration or phonons. From the absorption spectra, the energy level of the exciton polaron in CsPbBr_3_#(Tl/LA) NCs was the highest among all samples, leading to the strongest PL. In contrast, CsPbBr_3_#(Cl/LA) has a lower exciton polaron and considerably strong phonon interaction, such that the non-radiative decay pathway is more dominant, leading to weaker PL. The cubic structure of CsPbBr_3_#(Cl/LA) NCs has a higher degree of symmetry and smaller PbBr_6_ octahedral tilting, such that the excited state has a stronger interaction with phonons than that in the orthorhombic structure. In such a case, the non-radiative decay rate (the brown color path in Fig. 7) may overcome the radiative decay rate leading to poor PL characteristics. Recently, Yazdani et al. reported their finding on direct evidence of the coupling of exciton polarons and phonons on the degree of octahedral tilts in halide perovskites [72].

## 4 Conclusions

In this study, CsPbBr_3_ NCs powder with two dimensional shapes, so called nanoplatelets, with different crystal structures were obtained from synthesis using different pairs of ligands and antisolvents but without using OlAm as its co-ligand. Without OlAm, the formed NCs have a nanoplatelet shape. From the XRD data, the combination of toluene antisolvent and LA ligand yielded an orthorhombic crystal structure, whereas the other antisolvent-ligand pair used in this work yielded tetragonal and cubic crystal structures. Intense PL was observed in CsPbBr_3_ nanoplatelets with an orthorhombic crystal structure but not in CsPbBr_3_ NCs with tetragonal and cubic structures. This may be related to the formation of a small exciton polaron and weak interaction with lattice vibration or phonons due to a lower crystal symmetry caused by the presence of large octahedral tilting in orthorhombic CsPbBr_3_ NCs, which is in contrast to the cubic crystal structure. In addition, the high non-uniformity of the Br bonds on the crystal surface, as revealed by the XPS data for the orthorhombic CsPbBr_3_ NCs, may also hinder exciton diffusion and dissociation at the surface, leading to strong PL in the CsPbBr_3_ nanoplatelets prepared with toluene-LA. The present results may then provide additional insights into the role of the antisolvent and ligand to manipulate these perovskite crystal surface structures depending on the target applications, which may also relevant to other halide perovskites in general.

## CRediT authorship contribution statement

**Valdi Rizki Yandri:** Writing - review & editing, Writing - original draft, Methodology, Formal analysis, Data curation, Conceptualization. **Adhita Asma Nurunnizar:** Methodology, Data curation. **Rima Debora:** Methodology, Data curation. **Priastuti Wulandari:** Writing - review & editing, Validation, Supervision, Methodology. **Natalita Maulani Nursam:** Writing - review & editing, Validation, Supervision, Resources, Methodology. **Rahmat Hidayat:** Writing - review & editing, Writing - original draft, Validation, Supervision, Methodology, Investigation, Formal analysis, Conceptualization, Resources. **Efi Dwi Indari:** Methodology, Formal analysis, Data curation. **Yoshiyuki Yamashita:** Validation, Resources, Formal analysis, Data curation.

## Declaration of competing interest

The authors declare that they have no known competing financial interests or personal relationships that could have appeared to influence the work reported in this paper.
